# Employees’ healthy eating and physical activity: the role of colleague encouragement and behaviour

**DOI:** 10.1186/s12889-022-14394-0

**Published:** 2022-11-01

**Authors:** Anne van der Put, Lea Ellwardt

**Affiliations:** 1 Department of Sociology, Faculty of Social and Behavioural Sciences, Utrecht University, Padualaan 14, 3584 CH Utrecht, The Netherlands; 2 Institute of Sociology and Social Psychology, University of Cologne, Albert Magnus Platz, 50923 Cologne, Germany

**Keywords:** Healthy eating, Physical activity, Social influence, Social networks, Colleagues

## Abstract

**Background:**

Exercising and eating healthy are not just an individual choice, but influenced by family members, friends, or neighbours. Little is known, however, about colleagues, who are another important interpersonal influence. Many people spend many hours at work, surrounded by mostly the same colleagues, who could therefore significantly shape employees’ (un)healthy choices. We studied to what extent colleagues may play a part in one another’s eating and exercise behaviours by focusing on two pathways: colleagues can encourage a healthy lifestyle or act as role models whose behaviours can be observed and copied.

**Methods:**

We used the European Sustainable Workforce Survey, with data on 4345 employees in 402 teams in 113 organisations. We used network autocorrelation models, which resemble regression models, to study to what extent employee encouragement is related to fruit and vegetable consumption, and physical activity. Specific to this type of model is the inclusion of a network correlation parameter which allows for the outcome of an employee to be directly associated with the outcomes of their colleagues. In this way we tested whether colleagues’ behaviours were related to one another.

**Results:**

We found that employees were more likely to eat fruit and vegetables as well as engage in physical activity when their colleagues encourage a healthy lifestyle. Employees’ healthy eating behaviours were positively related to their colleagues’ fruit and vegetable consumption, while we found a negative correlation concerning physical activity.

**Conclusion:**

Overall, colleagues’ encouragement and own healthy behaviours have the potential to contribute to creating a culture of health in the workplace and support all employees in making healthy choices.

## Background

People’s lifestyle choices are shaped by their social environment. Partners, family members, friends, and neighbours have been shown to influence the extent to which people eat healthily and engage in physical activity [1–8]. As a result, researchers have increasingly looked beyond the individual level and also examined the social environment to understand how people make (un)healthy choices [9–11]. However, one relevant set of influential actors has received less attention: colleagues. This is surprising given that many adults spend the majority of their waking hours in the workplace, where they repeatedly encounter the same colleagues [12, 13]. The workplace is a social arena in which people interact often and on a daily basis [14], so that their shared time may surpass that spent with family and romantic partners [3]. In other words, colleagues should be included in an inquiry into the influence of social environments on (un)healthy behaviours. The present study examines to what extent colleagues may have a part in one another’s lifestyle choices, specifically healthy diets and physical activity.

Similarity between people’s lifestyle behaviours has been attributed to various mechanisms: homophily, shared environments, or more direct forms of influence [6]. In this article, we focus on the latter and argue for two potential ways in which colleagues can affect one another’s healthy behaviours: through encouraging each other to make healthy choices and by acting as role models. Firstly, via encouragement, colleagues can enhance each other’s motivation, increase their self-care and create a sense of shared responsibility for healthy choices [1, 2, 15]. Previous studies examined generic social support and social capital at the workplace, focussing more on trust between colleagues and solidarity [11, 13, 16, 17]. It is hard to see how this may enhance motivation for healthy choices, so unsurprisingly these studies found no association with healthy behaviours. By contrast, support specific to a behaviour has been suggested to be more predictive [18], also when it concerns employee healthy behaviour [19]. We therefore focus on encouragement from colleagues specific to healthy behaviours.

In serving as salient role models via their own lifestyle habits, colleagues can set norms and increase self-efficacy [7, 14, 20, 21]. Norms are a powerful driver of behaviour while self-efficacy determines the extent to which people enact a given behaviour. Hence, we additionally examine the role of colleagues’ own healthy behaviours. Previous studies tended to focus on employee reports of what their colleagues do, rather than what these colleagues actually do. Additionally, the focus has been largely on healthy behaviours in the workplace [9, 10, 20, 22] while we employ measures of eating and physical activity that also take place outside work. Both mechanisms, encouragement and role modelling, are expected to contribute making workplaces conducive to employee health and well-being, thereby creating a supportive environment for healthy behaviours [23, 24].

Previous studies involving colleagues’ roles in shaping lifestyle choices tended to (a) solely model formal membership in organisations rather than measure close proximity and the chance to meet [25], (b) incorporate group sizes too small to be conclusive [4] or (c) aggregate individual-level measures to group-level variables [13, 14], underestimating social influence from direct colleagues [26]. To overcome this research gap, we use unique multilevel data from the European Sustainable Workforce Survey – ESWS [27]. The ESWS comprise 4345 employees nested in 402 teams in 113 organisations in 9 European countries. Importantly, the data structure permits modelling which employees work together and thus have the potential to affect one another’s behaviours. We employ network autocorrelation models [28] to account for the interdependence of employees’ healthy behaviours within a team. Network autocorrelation models enable the direct association of employees’ outcomes with those of their colleagues, offering a better test for finding out how colleagues’ healthy behaviours are related.

We examine two different behaviours: healthy eating and physical activity. Together, they have great potential to improve health, and are often paired in lifestyle-related recommendations [21, 29]. Adults consume about a third of their daily calorie intake in the workplace and work impacts the extent to which employees engage in physical activity [12, 30]. For example, many office workers spend most of their working day sedentary. Eating and physical activity behaviours share a social component, for example, lunch is often eaten together in the workplace [17, 31], while differing substantially in exposure and ease of implementation. Since eating is typically more visible and occurs more frequently in the workplace than exercising, eating tends to be more prone to social influence [19]. Studying the two comparable yet different activities of eating and physical activity is meant to provide nuanced insights into the role of the social workplace for healthy choices.

## Explanatory mechanisms

### Perceived encouragement

Encouragement is the first way dealt with here in which colleagues can affect one another’s lifestyle behaviours [23, 32]. By talking about exercise and diets, both common topics of conversation in the workplace, employees can learn that their colleagues value a healthy lifestyle [20, 33]. This may translate into making healthy choices in three ways.

Firstly, encouragement can be considered positive reinforcement of desirable behaviour, which may enhance motivation [1]. If employees perceive their colleagues as endorsing a healthy lifestyle, they may deduce that it is important and be more likely to adopt such behaviours. This reflects a shared, generally implicit notion that if a person engages in behaviour that others approve of, they will approve of the person too [34].

Secondly, perceived encouragement could lead to a sense of responsibility towards those offering it. Not only does encouragement reflect what others find important, it also could create a sense of shared effort; employees could get the feeling that they do not want to let their well-meaning colleagues down by making unhealthy choices [1, 15]. In this way, colleagues keep one another accountable, for example by motivating the other to go for a lunch walk even when it is raining [7].

Thirdly, through encouraging healthy choices, colleagues demonstrate that they care about one another, as such choices are seen as something good [2, 7]. The sense of belonging that may result from this could increase self-care, such as eating better and engaging in sufficient physical activity [1, 16, 35].

Several studies have confirmed the notion that when employees perceive their colleagues as endorsing healthy behaviours, they are more likely to adopt such behaviours themselves. For example, in case colleagues encouraged healthy food choices, employees were more likely to participate in worksite health promotion programmes aimed at healthy diets. Similar results were found for physical activity programmes [19]. Colleague support has also been associated with exercise and diet, including increased fruit intake and physical activity [3, 29, 32, 36]. We thus hypothesise that the more an employee perceives colleagues as encouraging healthy eating, the more this employee will consume fruits (H1a) and vegetables (H1b). Similarly, the more an employee perceives colleagues as encouraging physical activity, the more this employee will engage in physical activity (H1c).

### Behaviour

The second way in which colleagues can affect one another’s lifestyle choices is through their own action. Colleagues have the potential to serve as important role models, whose behaviours can be observed, copied and influenced [14, 20]. At least three mechanisms may be at play here.

Firstly, colleagues’ choices to eat healthily and exercise, and how much, form a norm, which is considered a guide to appropriate behaviour [37]. Employees thus pay attention and learn what sorts of behaviours seem normal and expected, and, as proposed by social learning theory, follow this because they want to fit in with their co-workers [31, 38, 39]. Belonging to a group is considered one of the inherent human needs [35]. Commitment to group norms signals solidarity and earns approval. For example, employees reported feeling guilty if they ate unhealthy snacks while their colleagues chose healthy options [20]. Because adhering to norms may shape self-identity, behaviours at work can extend to other life spheres [38]. It should be noted, however, that norms can both support as well as undermine healthy choices. People may copy one another’s unhealthy choices in the same vein [7, 8, 12], for instance by regularly sharing chocolate cookies with their co-workers.

Secondly, colleagues’ actions may also enhance self-efficacy, that is people’s belief that they are able to behave in certain ways [21, 37]. Colleagues who work together may find themselves in the same situation: they take up similar positions in the organisation and engage in similar work tasks [14]. If employees notice that their co-workers manage to make healthy choices, this may signal to them that they are also capable of doing so. Similarly, self-determination theory [35] has argued that through this, the social environment nurtures employees’ intrinsic motivation to change their health behaviour. For example, seeing many colleagues bike rather than drive their two-mile to work may lead employees to ponder whether this is also something they could try. For self-efficacy, the comparison with similar others is key: frenetic colleagues (e.g., biking 10 miles per journey) may seem out of range and trigger feelings of demotivation [15, 30].

Thirdly, colleagues can engage in healthy behaviours in the workplace together. For example, employees can motivate each other to engage in physical exercise by participating in a sports class together [19]. Additionally, colleagues often have lunch together [40]. The behaviours that employees display together with their coworkers contribute to their overall healthy eating and physical activity, and may also extend to life outside work.

Both for healthy eating and exercising, previous studies have established that colleagues’ behaviours tend to relate to one another. Several authors demonstrated that employees who reported seeing their colleagues eat fruits and vegetables increased their intake of the same [9, 10, 20, 22]. Similarly, employees who reported their co-workers to engage more often in physical activity, for example by actively commuting to work, also engaged in more physical activity [10, 22, 41, 42]. Hence, we hypothesise that the more an employee’s colleagues eat fruits and vegetables, the more employees will consume fruits (H2a) and vegetables (H2b). Likewise, we expect employees to engage more in physical activity the more their colleagues exercise (H2c).

## Methods

### Data

To test our hypotheses, we use unique multilevel data from the European Sustainable Workforce Survey (ESWS) [27] This survey, first conducted in 2015-16, contains data on employees, their teams, and the organisations they worked for in nine European countries (Bulgaria, Finland, Germany, Hungary, the Netherlands, Portugal, Spain, Sweden, and the UK). Organisations were approached using stratified random sampling based on sector (manufacturing, health care, higher education, transport, financial services, and telecommunications) and size (1–99 employees, 100–249 employees, and 250 + employees). When the random sample did not yield enough participants in a stratum, referrals and personal connections were used to complement the selection. Within each organisation, a contact person (usually the human resources manager) decided on whether the organisation wanted to join the study. Upon a positive response, at least three teams were selected in consultation with the HR manager. All employees, and the manager, of those teams were addressed at work to fill out the survey in their own language. The HR manager provided information about the organisation as a whole. This data structure enabled us to construct the networks of employees who worked together in the same teams, which is necessary for our purposes.

Our study incorporates data from the second round of the ESWS, due to its detailed information on employees’ lifestyle choices, which is not included in the first round. Data for the second wave was collected in 2018-19. Organisations from the first round were invited to participate once again, and 13 new organisations also joined the study under the same selection and survey completion procedures as the first round. All participants provided written informed consent prior to filling out the survey. The response rate in the second wave was 89% among HR managers, 68% among team managers, and 54% among employees, resulting in a sample of 4345 employees working as part of 402 teams in 113 organisations.

Because our study addresses three different behaviours, we created three analytical samples: one for fruit consumption, one for vegetable consumption, and one for physical activity. For each analytical sample, we first excluded employees who had missing values on any of the variables included (N_fruit consumption_=1197, N_vegetable consumption_=1162 and N_physical activity_=1314). Most of these missing values were for the dependent variables.^1^ Since we are interested in employees’ networks we excluded employees who had no colleagues (N_fruit consumption_=37, N_vegetable consumption_=38 and N_physical activity_=39). Our final analytical samples were N = 3111, N = 3145 and N = 2992 for fruit consumption, vegetable consumption and physical activity, respectively.

### Measurements

The measurement of our dependent variables is similar to questions in the European Social Survey [43]. Fruit and vegetable consumption were measured by asking respondents how often they ate fruits, including frozen fruits but excluding juice, and how often they ate vegetables or salads, including frozen vegetables but excluding potatoes. For both fruit and vegetable consumption, response categories were 1 = three times a day or more, 2 = twice a day, 3 = once a day, 4 = less than once a day, but at least four times a week, 5 = less than four times a week, but at least once a week, 6 = less than once a week and 7 = never. Answers were recoded so that a higher score indicated higher fruit or vegetable consumption. Physical activity was measured by asking participants on how many of the last 7 days they walked quickly, did sports or other physical activity for 30 min or longer. This is in line with European recommendations for sufficient physical activity [44]. A higher score indicates engaging in physical activity on more days.

The independent variable, perceived encouragement of healthy behaviours by colleagues, was measured separately for healthy eating and exercise. For healthy eating, the item was “My colleagues encourage me to eat healthy food” and for exercise the item was “My colleagues encourage me to exercise frequently”. We created two variables, one for healthy eating encouragement and one for that of physical activity, as the correspondence principle holds that specific encouragement is likely more influential than generic encouragement [18]. Answer options ranged from (1) always to (5) never, and were reversed so that a higher score indicated more perceived encouragement.

We added several control variables to our analysis. Female, younger, and higher educated people reportedly eat healthier [45], while men, younger and higher educated tend to engage in physical activity more [46]. Therefore, our models controlled for gender (female = 1), age and years of education. We further controlled for self-rated health, as health and healthy behaviours are interlinked [43]. According to previous research, people with a partner tend to behave healthier than those without, so we added a control for having a partner [5].

Moreover, we included several variables related to the work context. Since employees who work more hours tend to have more contact with their colleagues, we included working hours. Employees who have been part of the same team for longer have had more opportunities to be influenced by their colleagues there, so we added tenure in years in the team. Physical activity in the workplace may also contribute towards total physical activity [47]. We therefore controlled for physical work demands, measured by how often employees’ duties involved standing, walking, or other physical activities. Additionally, how often employees worked from home — ranging from 1=(almost) never to 7 = four or five days a week was included, as employees tend to have less contact with their colleagues when doing home office often [19]. Whether the employer had worksite health promotion policies (WHP), and if employees used them, as this has been related to healthier behaviour [48, 49], and colleagues may affect one another’s lifestyle choices by participating in WHP together [19]. For fruit and vegetable consumption, this relates to catering or cafeteria menus offering healthy nutrition, and for physical activity, to sport facilities at work or a financial contribution towards a sport activity outside the workplace. Finally, we controlled for team size, sector, and country.

### Analyses

The pairwise correlations between the three outcome variables were low to moderate: *r*_vegetable consumption, physical activity_=0.12, *r*_fruit consumption, physical activity_=0.17 and *r*_fruit consumption, vegetable consumption_=0.49. We therefore fitted separate models for each outcome.

Because we expected employees’ healthy behaviours to be related, ordinary least squares regression models were not suitable: these models require observations to be independent – meaning that employees’ behaviours within a team may not correlate [26]. Indeed, a test using Moran’s *I* found autocorrelation for all the dependent variables: fruit consumption (χ^2^ = 129.39(1), *p* < 0.001), vegetable consumption (χ^2^ = 150.36(1), *p* < 0.001) and physical activity (χ^2^ = 21.22(1), *p* < 0.001). We thus used network autocorrelation models (also known as spatial lag models or network effects models), which account for the interdependency of observations, and are therefore commonly used in social network analysis.[26, 28] The model builds upon standard linear regression models and takes the form of Y = ρWY + βX + ε, where Y is the vector of the outcome variable, W the adjacency matrix denoting which observations are part of the network, X a matrix of independent variables, β the vector of associated coefficients and ε a vector with error terms. As can be seen from the equation, the network autocorrelation model allows for the outcome of an employee (Y) to be directly associated with the outcomes of their colleagues (ρWY). Due to the nested data structure, we know which employees work together in the same team, and these are the colleagues whose outcomes we consider.

A relevant feature of the network autocorrelation model is that it includes a parameter ρ, which estimates the strength of the network effect. The network effect tests whether employees’ behaviours are related to their colleagues. The parameter ρ is a measure of the degree to which an employee behaves similarly to their colleagues, and ranges between − 1 and + 1 [28]. For example, in the analysis for physical activity, ρ can be interpreted as the expected increase in the number of days an employee engages in physical activity if their colleagues increase their physical activity by an average of one day.

Central to a network autocorrelation model is the weight matrix W, which represents the influence mechanism in the network [28]. In our study, we constructed W in such a way that only employees who worked in the same team were seen as influencing one another’s behaviours, as these were direct colleagues. Hence, the resulting adjacency matrix recorded a link between observations if employees worked within the same team, but no link if they worked in different teams or organisations. To account for differences in team sizes, we employed row normalisation, a common practice when using network autocorrelation models [28]. In this procedure, each colleague has the same amount of influence, irrespective of team size. We created three separate weight matrices, to account for the different numbers of missing variables for our three outcomes.

We used a GS2SLS estimator for fitting the models because the alternative ML estimator reportedly produces biased estimates [50]. For the hypotheses on encouragement, we examined direct and spill-over effects. The direct effect estimated the association between encouragement and a dependent variable. However, spill-over effects may be present due to interdependency: if one employee changes her fruit consumption because her colleagues encourage her to do so, this also affects the fruit consumption of other colleagues based on the network effect. As explained earlier, we examined the network effort for the hypotheses on employee behaviour.

## Results

The descriptive results in Table 1 show that on average, employees scored 4.73 on fruit consumption and 4.95 on vegetable consumption. This translates into eating fruit and vegetables about once per day. On average, employees engaged in physical activity three days per week.

**Table 1:** Descriptive statistics

	Fruit consumption		Vegetable consumption		Physical activity		
	M	SD	M	SD	M	SD	Range
Fruit consumption	4.73	1.40					
Vegetable consumption			4.95	1.12			
Physical activity					2.92	2.21	0–7
Perceived encouragement eating	2.22	1.22	2.22	1.22			
Perceived encouragement physical activity					2.11	1.18	0–5
Female	0.58		0.58		0.57		
Age	43.97	11.40	44.00	11.38	43.64	11.37	19–77
Education in years	13.72	3.52	13.70	3.53	13.79	3.48	3–21
Health	3.86	0.72	3.86	0.72	3.86	0.72	1–5
Partner	0.74		0.74		0.74		
Children	0.50		0.50		0.50		
Work hours per week	39.63	9.77	39.60	9.80	39.65	9.74	0–60
Physical work demands	3.17	1.45	3.17	1.45	3.15	1.45	1–5
Tenure in years	8.81	8.67	8.84	8.70	8.63	8.63	0–49
Working from home	1.73	1.37	1.73	1.38	1.74	1.38	1–7
WHP							
Not available	0.46		0.46		0.44		
Available but not used	0.33		0.33		0.42		
Available and used	0.21		0.21		0.14		
Team size							
10 employees or less	0.39		0.38		0.39		
11–20 employees	0.23		0.25		0.25		
21 employees or more	0.38		0.37		0.35		
Sector							
Manufacturing	0.32		0.32		0.32		
Healthcare	0.26		0.26		0.25		
Higher education	0.22		0.22		0.23		
Transport	0.09		0.09		0.09		
Financial services	0.06		0.06		0.06		
Telecom	0.05		0.06		0.06		
Country							
UK	0.05		0.05		0.05		
Germany	0.06		0.06		0.06		
Finland	0.03		0.03		0.03		
Sweden	0.09		0.09		0.09		
The Netherlands	0.21		0.21		0.22		
Portugal	0.05		0.05		0.05		
Spain	0.04		0.04		0.04		
Hungary	0.18		0.18		0.18		
Bulgaria	0.29		0.29		0.27		
N	3111		3145		2992		

We first examined hypotheses 1a, 1b, and 1c, which associated more perceived encouragement by colleagues with increased fruit and vegetable consumption and physical activity. We find support for all three of these hypotheses as seen in Table 2. Perceived encouragement was positively correlated to fruit consumption (B = 0.071, *p* < 0.001), vegetable consumption (B = 0.052, *p* = 0.001), and physical activity (B = 0.086, *p* = 0.016). We furthermore found no significant spill-over for any of the three outcomes (fruit consumption: B = 0.034, *p* = 0.058, vegetable consumption: B = 0.016, *p* = 0.110 and physical activity: B=-0.029, *p* = 0.055). This means that behavioural changes due to higher perceived encouragement did not spill-over to other colleagues. These findings support our first set of hypotheses, associating perceived encouragement of healthy habits by colleagues with greater fruit and vegetable consumption and physical activity.

For our second set of hypotheses, we expected that the more an employee’s colleagues showed healthy behaviours, the more this employee would behave in healthy ways. We tested this using the network effect ρ, which related employees’ behaviours to that of their colleagues. The results on the network effects ρ in Table 2 suggest an association between colleagues’ fruit consumption (ρ = 0.329, *p* = 0.002) and vegetable consumption (ρ = 0.238, *p* = 0.024). If all colleagues raised their food consumption by one unit on average, this would lead to an increase of 0.329 in the employee’s fruit consumption and 0.238 in the employee’s vegetable consumption. Against our expectations, we found a significant negative network effect for physical activity (ρ=-0.449, *p* = 0.009). If colleagues were more physically active by one day on average, employees would decrease their own activity by about half a day.

Some of the results for the control variables are worth noting. Physical work demands were positively related to total physical activity (B = 0.074, *p* = 0.016). Additionally, WHP appeared to play no role in the extent to which employees eat fruits and vegetables, but was related to physical activity. Employees that had WHP aimed at physical activity (either sport facilities at work or a financial contribution towards a sport activity outside) available and used this, reported higher total physical activity (B = 0.327, *p* = 0.038).

**Table 2:** Unstandardised coefficients (B) and standard errors (SE) from the network autocorrelation models on health behaviours

	Fruit consumption		Vegetable consumption		Physical activity	
	B	SE	B	SE	B	SE
Perceived encouragement	0.071***	0.020	0.052**	0.016	0.086*	0.036
Network effect ρ	0.329**	0.108	0.238*	0.106	-0.449*	0.172
Female (ref = male)	0.237***	0.054	0.299***	0.043	-0.155	0.091
Age	0.010***	0.003	-0.000	0.002	0.013**	0.004
Education in years	0.015	0.009	0.037***	0.007	-0.023	0.015
Self-rated health	0.251***	0.034	0.173***	0.027	0.498***	0.058
Partner (ref = unpartnered)	0.159**	0.057	0.151**	0.045	-0.130	0.098
Children (ref = no)	0.019	0.050	-0.042	0.039	-0.238**	0.086
Work hours per week	-0.001	0.003	-0.004*	0.002	-0.008	0.004
Physical job demands	-0.008	0.018	-0.004	0.014	0.074*	0.031
Tenure in years	0.004	0.003	0.002	0.003	0.001	0.006
Working from home	-0.000	0.020	-0.002	0.015	0.052	0.033
WHP (ref = not available)						
Available, not used	-0.078	0.061	-0.044	0.048	-0.064	0.107
Available, used	0.064	0.072	0.057	0.057	0.327*	0.158
Team size (ref = 10 employees or more)						
11–20 employees	0.067	0.065	0.037	0.050	-0.060	0.108
21 employees or more	-0.011	0.059	0.016	0.046	-0.128	0.102
Sector (ref = manufacturing)						
Health care	0.018	0.078	-0.032	0.061	0.575***	0.143
Higher education	0.039	0.088	-0.048	0.068	0.427*	0.154
Transport	-0.117	0.104	-0.054	0.082	-0.070	0.173
Financial services	-0.094	0.113	-0.124	0.089	0.503*	0.206
Telecom	-0.033	0.119	-0.041	0.093	-0.351	0.205
Country (ref = Netherlands)						
UK	0.043	0.131	0.155	0.108	0.913***	0.235
Germany	-0.229	0.128	-0.077	0.097	0.205	0.208
Finland	-0.111	0.160	0.479**	0.141	1.206***	0.318
Sweden	-0.459***	0.124	0.082	0.083	0.531**	0.194
Portugal	0.307*	0.129	0.046	0.095	-0.598**	0.222
Spain	-0.008	0.135	-0.319**	0.117	1.445***	0.281
Hungary	-0.487***	0.119	-0.415***	0.093	0.324*	0.143
Bulgaria	-0.500***	0.106	-0.102	0.062	0.734***	0.161
Constant	1.403**	0.547	2.513**	0.527	1.522*	0.618
Pseudo R2	0.12		0.14		0.07	
N	3111		3145		2992	

Note: *p < 0.05, **p < 0.01, ***p < 0.001

### Sensitivity analyses

We performed several sensitivity analyses to gauge the robustness of our findings. This is especially relevant for the network effect, which is dependent on the construction of the weight matrix [28]. Firstly, instead of creating separate samples and weight matrices for each of the three dependent variables, we re-ran our analysis using a single sample and an identical weight matrix across all three models. The single sample was reduced to N = 2922, as it excluded employees whose information was incomplete on any of the three dependent variables. The results turned out highly robust for all hypothesised associations.

Secondly, we re-ran our analysis without using row normalisation, which assumed that every colleague had the same influence on an employee’s behaviour. Arguably, small teams offer fewer interaction partners than large teams, allowing for more frequent contact with every colleague. The results without row normalisation remained the same for perceived encouragement (H1a-1c) and network effect for vegetable consumption (H2b). We found a marginally significant network effect for fruit consumption (H2a: ρ = 0.086, *p* = 0.071) and a significant positive network effect for physical activity (H2c: ρ = 0.233, *p* = 0.049).

Thirdly, to further assess whether some colleagues affected one another more than others, we reconstructed the network using nomination data. In the survey, each employee had named up to three colleagues whom they meet outside work, and up to 3 colleagues with whom they enjoy working. Employees likely had more contact with these colleagues and could thus have been more influenced by them. The results for perceived encouragement remained stable (H1a-1c), while all three of the network effects became insignificant (H2a-2c). However, this null effect, was likely caused by poor statistical power, as those networks were extremely sparse [26].

Fourthly, to assess whether WHP may play a role in the extent to which colleagues affect each other’s behaviours, we re-ran our analyses without the control variable for availability and use of WHP. For fruit consumption and physical activity our results remained robust, but in the case of vegetable consumption we found a marginally significant network effect (*p* = 0.059).

Lastly, to assess whether results could be country- or sector-specific, we performed jack-knife procedures excluding either one country or one sector at a time [51]. The findings stayed the same concerning perceived encouragement for fruit consumption, vegetable consumption and physical activity (H1a-1c). The findings also turned out similar for the network effects on fruit consumption (H2a). By contrast, the network effects remained weaker for both vegetable consumption and physical activity (H2b-2c): the effects remained unaltered in terms of direction but failed to reach statistical significance in some of the subsamples. This suggests the impact of colleagues’ behaviours may differ per sector and country.

## Discussion

The aim of this study was to gauge colleagues’ effects, if any, on one another’s healthy behaviours. While previous studies demonstrated that partners, family members, friends and neighbours influence the extent of people’s healthy eating and exercising, the role of colleagues has remained understudied [1–8]. This is surprising, considering the amount of time many adults spend at work, frequently interacting with the same colleagues [12, 14]. We studied whether colleague encouragement and behaviour could be associated with the extent to which employees eat fruit and vegetables and engage in physical activity [23, 24]. We used the European Sustainable Workforce Survey [27], with data on employees nested in teams, to allow us to reconstruct which colleagues worked together and could thus directly affect one another.

We found that, as expected, the more employees perceived their colleagues to encourage them to behave in healthy ways, the higher the employee’s fruit and vegetable intake, and the more the employee was physically active. This result is in line with previous studies [3, 29, 32, 36]. Perceived encouragement may relate to healthy behaviours in several ways – e.g. through positive reinforcement [1], instilling a sense of responsibility [15], and creating a sense of belonging [16] – all of which may increase self-care and enhance motivation. Some studies looked at generic social support or social capital and found no effect [11, 13, 16, 17] while our findings suggest that support specific to the behaviour does matter.

Our network models also showed that employees’ healthy eating may follow from colleagues’ fruit and vegetable consumption, as colleagues may represent salient role models whose behaviour sets the norm about what could be expected based on social learning theory [39]. Moreover, observing behaviour from colleagues may increase the employee’s self-efficacy, by demonstrating the ability to, for example, bring a healthy snack to work rather than buying from the vending machine [20, 21]. Since employees consume about a third of their daily calories in the workplace, this is an important setting to promote healthy eating [12]. Previous studies mainly gauged healthy eating in the workplace and found similar results [9, 10, 20, 22]. Our measure of healthy eating comprised fruit and vegetable intake in general, thus also outside the workplace. Our results suggest that colleagues’ influence extends to private and leisure settings.

As opposed to our expectations, we found a negative correlation between employees’ and colleagues’ physical activity. This result is not yet conclusive, since the sensitivity analysis indicated a positive trend when allowing for the influence of colleagues to vary with team size. Previous studies did find an association between employees’ and colleagues’ physical activity [10, 22], but they focused mainly on employees’ perceptions of colleagues’ behaviour instead of their actual behaviours, which may arguably matter more. One explanation for our negative result may be that physical activity typically takes place outside work, where it is hardly visible to colleagues. Eating at work takes place daily and usually happens together with colleagues, whereas physical activity behaviours occurs less frequently, making it less prone to social influence [19]. Some employees may participate in group sports classes at the workplace together with their colleagues, but this is arguably a small group. Additionally, in our sample WHP initiatives aimed at healthy eating were more often used than programmes promoting physical activity, which may also mean eating behaviour of colleagues is more visible. To be effective, norms should be specific to the situation [52–54], hence norms on dieting can be supported more easily via observation of others in the workplace, a setting which often includes eating meals [19]. Moreover, extremely athletic colleagues could demotivate others by giving them the impression that this level of physical activity is out of reach for them [15, 30].

Of further interest is to note that employees with more physical work demands reported higher physical activity, which could be shared by colleagues who work in a similar job. These employees may already feel that they are active enough during the working day, although previous research has shown that occupational physical activity is no substitute for leisure-time physical activity [47]. Finally, even though employees often engage in WHP together with their colleagues [19], which could be one of the mechanisms through which colleagues’ lifestyle choices relate to each other, results showed the influence of colleagues extends beyond shared WHP use. The exception here concerned vegetable consumption, which we no longer found statistically significant when removing WHP from our analyses, suggesting that the impact of colleagues eating vegetables could take place during shared lunch in the healthy worksite cafeteria. Nevertheless, these findings show that colleagues also matter for employees that do not participate in WHP.

### Limitations and suggestions for future research

Several limitations of our study are worth noting. Firstly, the cross-sectional research design was unable to separate selection from influence processes [2]. However, it seems unlikely that employees choose to work in a formal work team based on the healthy behaviours of colleagues. Although the extent to which healthy behaviours are the norm may differ among occupations, we addressed this variation by including control variables for education and sector. Future research would benefit from using longitudinal data to examine influence processes over time. As argued by self-determination theory [35], individuals internalise cues from their environment to shape their intrinsic motivation. It would thus be interesting to study how long it takes a new employee to adapt to current workplace health norms.

Secondly, as noted in the robustness analyses, the network effect is dependent on the construction of the weight matrix [28]. We based the weight matrix on colleagues working in the same department. Not all employees within each department filled out the questionnaire and some employees were more likely to lack information on the eating and physical activity variables as we have shown. Especially in large departments, employees may not have had contact with all their colleagues. Data on complete networks would have been desirable. We addressed this potential shortcoming by examining several alternative specifications of the weight matrix, which provided mostly consistent results.

Thirdly, the measure of physical activity was very general and addressed any type of physical activity in the past week. This could range from moderate activities such as walking the dog or cycling to work to more extreme sports such as mountain biking or running half marathons. The fact that these activities may be very different could further explain why we did not find a network effect. Similarly, our measures for fruit and vegetable consumption could have been more informative, for example by highlighting how large a portion is or by following the WHO guideline to eat at least 400 g of fruit and vegetables per day [55]. However, other studies, such as the European Social Survey [43], used the same measures, making our findings comparable.

Fourthly, in focussing on the role of perceived encouragement and colleague behaviour, we left out other mechanisms that may explain how workplace social relations affect healthy eating and physical activity behaviours. For example, we have tended to focus on positive influences, but also processes of peer pressure, social control and stigmatisation (e.g. fat shaming) could impact the healthy choices employees make [10, 56]. In addition, our measure for perceived encouragement was very general. A more detailed measure may provide richer insights. In order to paint a complete picture of the role colleagues may have in each other’s healthy habits, future studies should address this too.

Finally, some more information on the context and environment in which employees work together with their colleagues would have provided more insights. For example, it may be easier for colleagues to exercise together at work when there are showers present, or when the office is close to a park where they can go for a lunch walk together. We addressed this with the data we had available by including a control variable for WHP, but it would be good if future studies pay more attention to the environment. This may also provide insights in whether colleagues are more or less influential depending on the other options present for making healthy choices in the workplace.

### Strengths and implications

Among our study’s strengths were the focus on both colleagues’ encouragement and actual behaviours, addressing encouragement specific to the behaviour rather than generic social support and examining behaviours that also take place outside the workplace. Furthermore, our study is one of the first to address the role of co-workers’ behaviours using a network approach incorporating direct colleagues. This allowed for a finer grained analysis than the aggregation of individual-level measures or relating employees who may not work in close proximity. This study thus represents an important first step, showing that it is promising for managers and public health policy makers to incorporate workplace social relations to promote healthy behaviours.

The implications of our study relate to the realisation that when designing health interventions, it is important to incorporate the social work environment alongside other social actors such as partners, family members, and friends. We showed that colleagues are relevant sources of social support when it comes to healthy behaviours and may act as role models. In stimulating employees to make healthy choices, organisations can make use of mentors or health champions, which are employees who have adopted a healthy lifestyle themselves and help their colleagues to do so too [57]. For worksite health promotion activities, it is also important to draw on the positive influence colleagues could have in helping one another make healthy choices. Crucially, not only do colleague encouragement and behaviours contribute to creating a culture of health in the workplace [23], they also indirectly support the entire work population, including those not using dedicated programmes in the workplace.

## Conclusion

Our study showed that employees are more likely to eat fruit and vegetables as well as engage in physical activity when their colleagues encourage a healthy lifestyle. Employees’ healthy eating behaviours were positively related to their colleagues’ fruit and vegetable consumption, while we found a negative correlation concerning physical activity. Overall, colleagues’ encouragement and own healthy behaviours have the potential to contribute to creating a culture of health in the workplace and support all employees in making healthy choices. These results show that companies seeking to promote healthy life styles may supplement their corporate policies with a socially supportive infrastructure in the workplace.

## Data Availability

The dataset supporting the conclusions of this article is available in the DataCite repository, DOI: 10.24416/UU01-87ECE1. [27].

## Footnotes

1 We checked whether certain employees were more likely to have incomplete information for the dependent variables by regressing whether the dependent variable was missing on all explanatory variables. Higher educated and employees who worked more hours were less likely to have missing information for fruit consumption. Those with a partner were less likely to have missing information for vegetable consumption. Higher educated, younger and employees who used were less likely to have missing information for physical activity.

## List of abbreviations

ESWS: European Sustainable Workforce Survey
